# Bone mineral density after exercise training in patients with chronic kidney disease stages 3 to 5: a sub-study of RENEXC—a randomized controlled trial

**DOI:** 10.1093/ckj/sfad287

**Published:** 2023-11-21

**Authors:** Vaida Petrauskiene, Matthias Hellberg, Philippa Svensson, Yunan Zhou, Naomi Clyne

**Affiliations:** Lund University, Skåne University Hospital, Faculty of Medicine, Department of Clinical Sciences Lund, Nephrology, Lund, Sweden; Lund University, Skåne University Hospital, Faculty of Medicine, Department of Clinical Sciences Lund, Nephrology, Lund, Sweden; Lund University, Skåne University Hospital, Faculty of Medicine, Department of Clinical Sciences Lund, Nephrology, Lund, Sweden; Lund University, Skåne University Hospital, Faculty of Medicine, Department of Clinical Sciences Lund, Nephrology, Lund, Sweden; Lund University, Skåne University Hospital, Faculty of Medicine, Department of Clinical Sciences Lund, Nephrology, Lund, Sweden

**Keywords:** bone mineral density, chronic kidney disease, dual energy X-ray absorptiometry, exercise training, physical activity

## Abstract

**Background:**

We evaluated the effects of 12 months of exercise training on bone mineral density (BMD) in patients with chronic kidney disease (CKD) stages 3–5 not on kidney replacement therapy (KRT).

**Methods:**

A total of 151 patients were randomized to 12 months of either balance or strength training, both together with endurance training. Some 112 patients completed and 107 (69 men, 38 women) were analysed, with a mean age 66 ± 13.5 years and 31% having diabetes. The exercise training was self-administered, prescribed and monitored by a physiotherapist. Total body, hip and lumbar BMD, T score and Z score were measured at baseline and after 12 months using dual energy X-ray absorptiometry.

**Results:**

Both groups showed increased physical performance. The prevalence of osteoporosis and osteopenia was unchanged. The strength group (SG) decreased total body BMD (*P* < .001), the balance group (BG) increased total body T score (*P* < .05) and total body Z score (*P* < .005). Total body ΔT score was negative in the SG and unchanged in the BG (*P* < .005). Total body ΔZ score was negative in the SG and positive in the BG (*P* < .001). The proportion of progressors measured by ΔT (*P* < .05) and ΔZ scores (*P* < .05) was significantly lower in the BG compared with the SG. In multivariate logistic regression analysis, belonging to the BG was the only factor with a lower risk of deterioration of total body BMD, T and Z scores.

**Conclusions:**

Twelve months of balance training together with endurance training seemed to be superior to strength training in maintaining and improving BMD in patients with CKD not on KRT.

KEY LEARNING POINTS **What was known:**

- Patients with chronic kidney disease (CKD) exhibit a considerable risk of skeletal fractures as the result of a large spectrum of kidney disease–related bone disorders in addition to a variety of other factors including age-related osteoporosis.
- Physical activity and in particular resistance training have been shown to be an effective and safe intervention positively influencing bone mineral density (BMD) and bone turnover in the general population.
- Several studies have evaluated the effects of different exercise training programs on changes in BMD in patients on dialysis. However, the association between changes in bone parameters and different types of exercise training has not been examined in non-dialysis-dependent patients with CKD.

**This study adds:**

- This study is the first head-to-head study comparing the effects of strength or balance training, both in combination with endurance training, on bone health parameters in patients with CKD.
- Balance training seemed to be superior to strength training, both in combination with endurance training, in maintaining and improving bone health in non-dialysis-dependent patients with CKD.
- This study shows that balance training is an effective form of exercise training in patients with CKD and can contribute to preserving bone health and maintaining good balance function, both important outcomes considering the high risk of falls in the elderly.

**Potential impact:**

- Considering the positive results of our study on physical performance and bone health, balance training should be incorporated in prescriptions for physical activity as well as osteoporosis preventive training programs aiming not only to improve balance and minimize fall risk, but also to ameliorate bone health parameters in patients with CKD.

## INTRODUCTION

Disturbances in mineral and bone metabolism occur early during chronic kidney disease (CKD) which in combination with traditional risk factors for bone fragility, lead to impaired bone quality and quantity and increased fracture risk [1–3]. Two studies in patients on dialysis found a higher incidence of fractures in the hip and vertebrae compared with the general population [4, 5]. There are few studies on bone mineral density (BMD) in patients with CKD and most are cross-sectional. Change in BMD is a slow process and may take 2–3 years to become significant [1, 6], although the decline in BMD might be accelerated in patients with CKD resulting in a higher rate of bone loss [1]. KDIGO suggests testing BMD in patients with CKD stages 3–5D who have evidence of CKD-MBD (mineral and bone disorder) and/or risk factors for osteoporosis, for fracture risk assessment if results will impact treatment decisions [7].

Exercise training and in particular resistance or strength training has been shown to be an effective intervention with positive effects on BMD and bone turnover in individuals without CKD [8, 9]. In patients on haemodialysis 24 weeks of intradialytic resistance training improved BMD and the T score in the femoral neck [10]. In a study in patients on peritoneal dialysis, 6 months of exercise had no effect on BMD at any of the skeletal sites [11]. To our knowledge the effects of exercise training on BMD have not been studied in patients not on kidney replacement therapy (KRT) with CKD stages 3–5, nor are there any studies with a duration of 12 months.

The primary hypothesis of the RENEXC (RENal EXerCise) study was to investigate whether 12 months of strength training was superior to balance training, both together with endurance training, in patients with CKD stages 3–5 not on KRT. Physical performance was the primary outcome and improved in both groups, with no between-group differences [12].

The hypothesis in this pre-determined sub-study of the RENEXC trial was that strength training would be superior to balance training in preserving or improving BMD. The aim was to investigate the effects of strength and balance training, respectively, on (i) osteoporosis and BMD and (ii) the relationships between physical performance and BMD.

## MATERIALS AND METHODS

### Study design

This study is a prespecified sub-study of the RENEXC trial (www.ClinicalTrials.gov; NCT02041156), a prospective randomized controlled exercise training trial, with an intervention period of 12 months. There were no changes to protocol after the trial started. Complete study design and primary analysis of RENEXC data have been reported previously [12]. Some information on study design and methods is repeated here for clarity.

The inclusion criteria were incident and prevalent patients at the Department of Nephrology in Lund, Skåne University Hospital with an estimated glomerular filtration rate (GFR) <30 mL/min/1.73 m^2^, age ≥18 years, all kidney diseases and any number of comorbidities. Exclusion criteria were orthopaedic impediment, severe neurological dysfunction, inability to understand patient information and estimated start of KRT within 12 months of study start.

The study was approved by the Regional Ethical Review Board in Lund (registration number 2011/369) and adhered to the Helsinki Declaration. All participants gave informed consent prior to inclusion after having received written and oral information.

### Intervention

A total of 151 patients were randomized to either strength or balance training, both together with endurance training. The exercises were individually prescribed, monitored by physiotherapist, and self-administered at home or at a nearby gym. The Borg Rating of Perceived Exertion scale (RPE) was used to prescribe exercise intensity and to monitor progress [13]. The prescribed weekly exercise duration was 150 min, comprising 60 min per week of endurance training at a RPE of 13–15 for both groups combined with 90 min per week of either strength or balance training at a RPE of 13–17 per exercise set. Strength training mainly comprised exercises of the limbs performed in a sitting or supine position using an exercise machine, dumbbells or weighted cuffs. Balance training comprised exercises performed in a standing position engaging the whole body and core muscles.

All patients were tested at baseline (T0) and after 12 months (T12).

Measures of physical performance were the primary outcomes. Predetermined secondary outcomes were changes in BMD and the relationship between bone parameters and physical performance.

### Physical function

Overall endurance was tested with the 6-min walk test [14]. Muscular endurance and fatigability in the proximal leg muscles were tested with the 30-s sit-to-stand test [15]. Balance was tested with functional reach [16, 17]. Neuromuscular function and strength in the lower extremities were tested with isometric quadriceps strength [16, 17].

### Dual-energy X-ray absorptiometry

BMD was measured using dual-energy X-ray absorptiometry (DEXA) at the Department of Diagnostic Radiology, Skåne University Hospital, which is accredited by WSEDAC according to ISO 15189:2012. The hospital changed from Lunar Prodigy to Lunar iDEXA during the study period. Lunar iDEXA is an upgrade from Luna Prodigy. The analysis software is the same as Lunar Prodigy. Thirty-two patients were analysed with Lunar iDEXA.

### Comorbidity score

Comorbidity was assessed with the Davies Comorbidity Score [18] at baseline.

### Definition of osteoporosis

The World Health Organization (WHO) definitions of T score, Z score, osteopenia and osteoporosis were used in this study. The T and Z scores were calculated for the lumbar spine, hip and total body in each patient. Osteopenia is defined as a T score between –1.0 and –2.5; osteoporosis is defined as a T score at or below –2.5 [19, 20].

To evaluate between-group changes after the study period ΔBMD, ΔT score and ΔZ score values (T12 value – T0 value) were calculated. These results were used to define progressors and non-progressors.

### Laboratory analyses

Plasma 25 hydroxyvitamin D was analysed using liquid chromatography–mass spectrometry. GFR was measured using iohexol clearance. Albumin was analysed using immunoturbidimetric method on Cobas. All laboratory analyses were performed at the Department of Clinical Chemistry, Laboratory Medicine Skåne, which is accredited by SWEDAC (ISO 15189:2012).

### Randomization

Patients were randomized using ProcPlan in SAS (SAS Institute, Cary, NC, USA). The statistician generated the random allocation sequence, the nephrologist enrolled patients and the physiotherapist assigned participants to intervention. Patients were included and allocated sequential treatment according to a list that only the research physiotherapist had access to. Both interventions comprised endurance training. The difference between the treatment arms was that one group was allocated strength training and one group balance training.

### Statistical analyses

Statistical analyses were performed using the SPSS for Windows software program version 24.0. Per-protocol analysis was employed including only patients who completed the treatment originally allocated. Variables were expressed as frequencies and percentages for discrete factors. Continuous factors are presented as means ± standard deviations or medians (minimum and maximum). The two-tailed chi-square test was used for categorical variables and the two-tailed Student's *t*-test or Mann–Whitney test for continuous variables, according to data distribution. The paired sample T-test was used for parametric variables and McNemar's test for paired nominal data.

To identify clinical factors and laboratory changes that could affect progression of changes in bone mass, the odds ratio obtained from univariate and multivariate binary logistic regression was used where completers from both groups were pooled and analysed together as there were no between-group differences for measurements of physical performance. For all comparisons a *P*-value <.05 was considered significant.

## RESULTS

**Figure 1:**
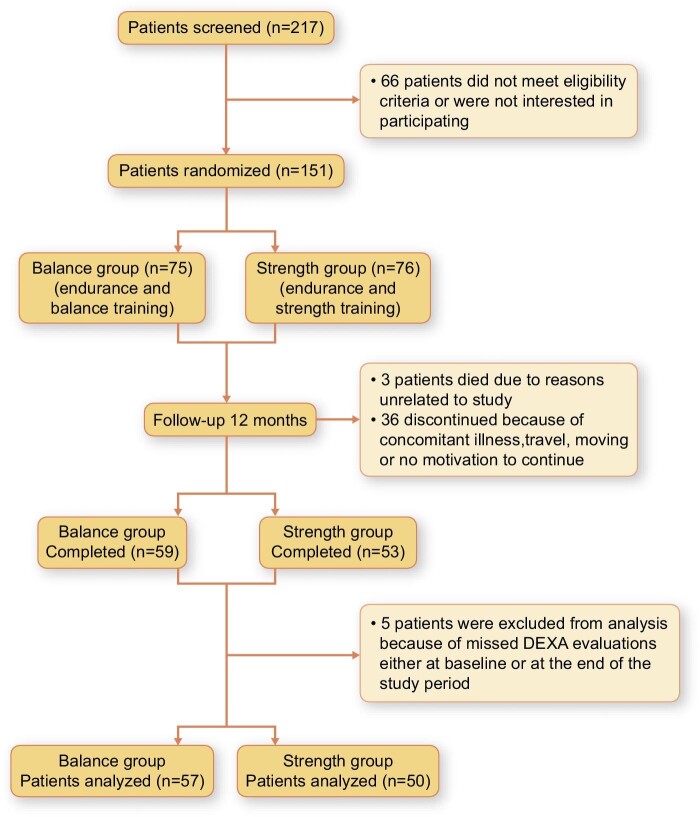
CONSORT Flow diagram for the RENEXC trial.

**Figure 2:**
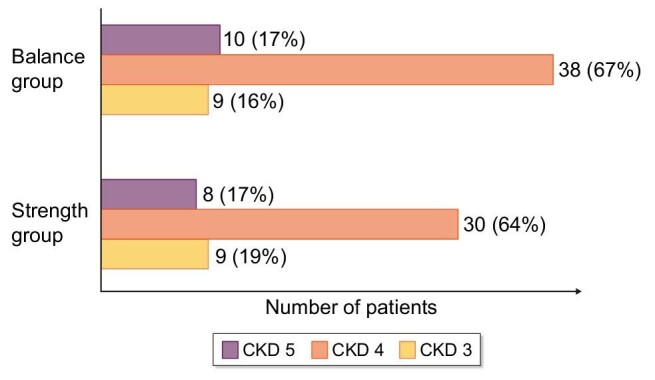
Distribution of CKD stages within training groups.

A total of 217 patients were screened and 151 patients were randomized. Some 112 patients completed the whole training period, of whom 5 patients were excluded because of missing DEXA evaluations. Consequently, results from 107 (69 men, 38 women) patients (mean age 66 ± 13.5 years, measured GFR 22.7 ± 8 mL/min/1.73 m^2^) were analysed. Fifty patients (34 men, 16 women) participated in the strength group, and 57 (35 men, 22 women) in the balance group. The Consolidated Standards of Reporting Trials (CONSORT) flow diagram is presented in Fig. 1. Some clinical characteristics and laboratory data are presented in Tables 1 and 2. The CKD staging in each group is presented in Fig. 2.

**Table 1:** Some clinical characteristics at baseline.

	*n*	Strength group	*n*	Balance group	*n*	Whole group
Age, years	50	67.6 ± 11.4	57	65.6 ± 15.2	107	66.5 ± 13.5
Male, *n* (%)	50	34 (68.0)	57	35 (61.4)	107	69 (64.5)
Weight, kg	47	86.3 ± 19.0	57	80.5 ± 15.5	104	83.1 ± 17.4
Height, m	47	1.7 ± 0.1	57	1.7 ± 0.1	104	1.7 ± 0.1
BMI, kg/m^2^	47	27.5 (19.6–52.3)	57	27.4 (20.9–38)	104	27.4 (19.6–52.3)
Medications, *n* (%)						
Diuretics^a^	49	31 (62)	57	40 (70)	106	71 (67)
PPI	49	14 (28)	57	13 (23)	106	27 (25)
Cinacalcet	49	1 (2)	57	2 (3.5)	106	3 (3)
Active vit D	49	31 (63)	57	35 (61)	106	66 (62)
Native vit D	49	31 (63)	57	36 (63)	106	67 (63)
Calcium-based phosphate binders	49		57		106	
Calcium carbonate		16(33)		22(39)		38(36)
Calcium acetate		1 (2)		1 (2)		2 (2)
Non-calcium-based phosphate binders	49		57		106	
Sevelamer		6 (12)		1(2)		7 (6.5)
Lanthanum		2 (4)		2(3.5)		4 (4)
Comorbidity, *n* (%)						
Malignancy	49	8 (16)	57	8 (14)	106	16 (15)
Ischaemic heart disease	49	10 (20)	57	12 (21)	106	22 (20)
Peripheral vascular disease	49	9 (18)	57	9 (16)	106	18 (17)
Left ventricular dysfunction	49	7 (14)	57	5 (9)	106	12 (11)
Diabetes mellitus	49	18 (37)	57	15 (27)	106	33 (31)
Systemic collagen vascular disease	49	3 (6)	57	3 (5)	106	6 (6)
Other (e.g. hypertension)	49	53 (71)	57	43 (75)	106	78 (73)

Data are presented as mean ± SD or median (minimum–maximum).

^a^Ten patients received hydrochlorothiazide; all others received loop diuretics.

BMI, body mass index; PPI, proton pump inhibitors; vit D, vitamin D.

**Table 2:** Some laboratory data at baseline and after 12 months of training.

	Strength group			Balance group			Whole group		
Characteristics	T0	T12	*P*	T0	T12	*P*	T0	T12	*P*
P-calcium (mmol/L)	2.3 ± 0.1	2.3 ± 0.1	.56	2.3 ± 0.1	2.3 ± 0.1	.5	2.32 ± 0.11	2.30 ± 1.40	.377
P-phosphate (mmol/L)	1.1 ± 0.2	1.1 ± 0.3	.12	1.2 ± 0.2	1.2 ± 0.3	.162	1.1 ± 0.2	1.2 ± 0.3	**.035**
P-PTH (pmol/L)	10 (5.4–50)	13 (3.6–59)	**.048**	11 (3–133)	13.5 (1.8–48)	.09	11 (3–133)	13 (1.8–59)	**.011**
P-ALP (μkat/L)	1.3 (0.8–2.6)	1.3 (0.8–45)	.40	1.2 (0.7–2.5)	1.3 (0.6–3.1)	.47	1.3 (0.66–45)	1.3 (0.65–9.5)	.449
P-25(OH)D (nmol/L)	54 (29–103)	64 (33–113)	.191	67 (13–125)	68 (22–186)	.962	62 (13–125)	67 (22–186)	.471
P-albumin (g/L)	36.3 ± 4.1	36.0 ± 11.8	.658	37.7 ± 3.2	36.9 ± 5.6	.232	37.0 ± 3.75	36.5 ± 4.78	.204
P-CRP (mg/L)	3.2 (0.5–27)	2.8 (0.5–34.0)	.992	2.1 (0.5–58)	2.85 (0.5–37)	.118	2.7 (0.5–58)	2.8 (0.5–37)	.270
Base excess (mg/L)	–1.4 (–6.8 to 9.1)	–0.5 (–5.6 to 3.8)	.331	–1.2 (–7.7 to 8.6)	–1.35 (–9.2 to 7.7)	.218	–1.2 (–7.7 to 9.1)	–0.8 (–9.2 to 7.7)	.123
mGFR (mL/min/1.73 m^2^)	22.6 ± 8.7	21.1 ± 9.6	**.004**	22.8 ± 7.9	20.5 ± 7.6	**.001**	22.7 ± 8.2	20.8 ± 8.4	**.001**

Data are presented as mean ± SD or or median with minimal–maximal values.

T0, at the baseline; T12, after 12 month of training; mGFR, measured glomerular filtration rate; P, plasma; CRP, C-reactive protein; PTH, parathyroid hormone; ALP, alkaline phosphatase; 25(OH)D- 25 hydroxycholecalciferol.

A *p* value < 0.05 was accepted as the level of significance.

Table 3 shows the effects of 12 months of exercise training on physical performance. Both groups improved overall endurance, muscular endurance and strength and balance significantly, with no between-group differences.

**Table 3:** Effects of 12 months of exercise training on physical performance.

	Strength group				Balance group				Whole group			
	*n*	T0	T12	*P*	*n*	T0	T12	*P*	*n*	T0	T12	*P*
6-MWT (m)	43	404 ± 108	444 ± 129	**.0001**	53	432 ± 131	470 ± 134	**.004**	96	430 ± 114	459 ± 131	**.0001**
30-STS (*n*)	47	11.3 ± 5.8	12.8 ± 6.8	**.007**	57	11.7 ± 6.0	13.07 ± 7.8	**.002**	104	11.4 ± 5.7	12.8 ± 7.4	**.0001**
Functional reach (cm)	50	32.1 ± 7.7	35.3 ± 6.8	**.004**	55	33.6 ± 8.4	35.7 ± 8.0	**.007**	105	32.8 ± 8.1	35.6 ± 7.5	**.0001**
Isometric quadriceps strength (kg × cm)												
Right	50	1165 ± 365	1306 ± 390	**.001**	56	1119 ± 374	1209 ± 435	**.006**	106	1141 ± 1369	1254 ± 416	**.0001**
Left		1183 ± 383	1271 ± 458	**.033**		1077 ± 396	1193 ± 445	**.001**		1127 ± 391	1229 ± 450	**.0001**

Data are presented as mean ± SD.

T0, at baseline; T12, after 12 months of exercise training; 6-MWT, 6-min walk test; 30-STS, 30-s sit-to-stand test.

A *p* value < 0.05 was accepted as the level of significance.

### Osteoporosis and osteopenia

**Figure 3:**
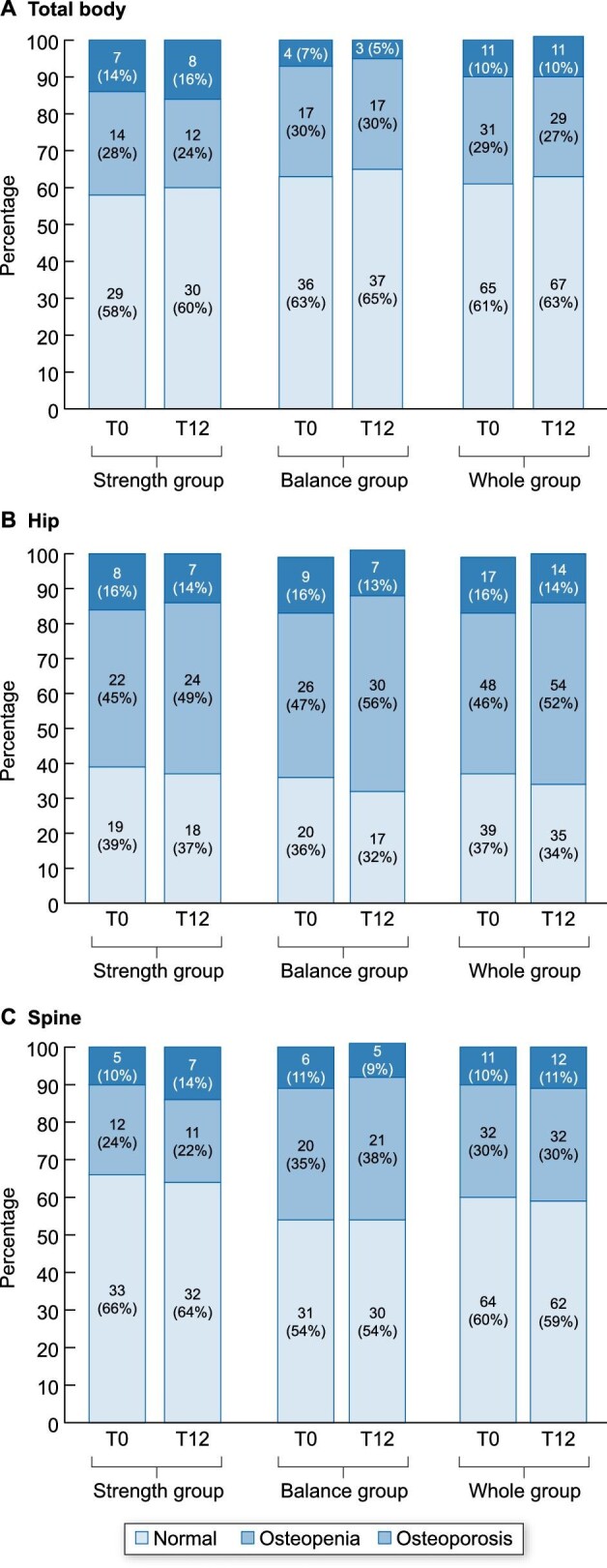
Prevalence of osteoporosis at baseline and after 12 months of exercise training. Data are presented as *n* (%). There were no statistically significant changes after 12 months of exercise training (*P* > .05).

The prevalence of osteoporosis and osteopenia was unchanged after 12 months of exercise training in the spine, hip and total body (Fig. 3). There were no between-group differences at baseline or after 12 months.

### Bone mineral density, T-score and Z-score

Values for BMD, T score and Z score at baseline and after 12 months of exercise training are shown in Table 4. The strength group showed a decrease in total body BMD (*P* = .001); all other measures were unchanged. The balance group showed an increase in total body T score (*P* = .035) and total body Z score (*P* = .003); all other measures were unchanged. There were no statistically significant between-group differences in any of the DEXA measurements at baseline or after 12 months of training.

**Table 4:** BMD, T score and Z score in the strength group, balance group and whole group at baseline (T0) and after 12 months of exercise training (T12).

	Strength group			Balance group			Whole group		
	T0	T12	*P*	T0	T12	*P*	T0	T12	*P*
Lumbar spine									
BMD (g/cm^2^)	1.226 ± 0.297	1.226 ± 0.304	.94	1.204 ± 0.311	1.198 ± 0.270	.16	1.215 ± 0.303	1.211 ± 0.285	.39
T score	0.23 ± 2.42	0.18 ± 2.44	.44	–0.05 ± 2.50	–0.19 ± 2.19	.62	–0.01 ± 2.30	–0.02 ± 2.31	.81
Z score	0.86 ± 2.09	0.85 ± 2.11	.89	0.46 ± 2.45	0.35 ± 2.10	.27	0.64 ± 2.23	0.59 ± 2.11	.46
Hip									
BMD (g/cm^2^)	0.920 ± 0.208	0.910 ± 0.201	.07	0.902 ± 0.206	0.894 ± 0.217	.12	0.911 ± 0.206	0.902 ± 0.21	**.016**
T score	–1.10 ± 1.40	–1.12 ± 1.39	.67	–1.25 ± 1.45	–1.28 ± 1.52	.84	–1.17 ± 1.42	–1.20 ± 1.45	.65
Z score	–0.26 ± 1.18	–0.22 ± 1.15	.45	–0.55 ± 139	–0.54 ± 1.47	.93	–0.41 ± 1.29	–0.39 ± 1.34	.64
Total body									
BMD (g/cm^2^)	1.153 ± 0.151	1.141 ± 0.153	**.001**	1.160 ± 0.151	1.157 ± 0.173	.57	1.156 ± 0.150	1.149 ± 0.164	**.032**
T score	–0.39 ± 1.56	–0.48 ± 1.56	.14	–0.31 ± 1.66	–0.20 ± 1.70	**.035**	–0.35 ± 1.61	–0.33 ± 1.64	.65
Z score	0.07 ± 1.19	0.01 ± 1.22	.052	0.05 ± 1.62	0.311 ± 1.62	**.001**	0.06 ± 1.43	0.16 ± 1.45	.037

Data are presented as mean ± SD.

A *p* value < 0.05 was accepted as the level of significance.

When stratified for CKD stage, patients with CKD stage 4 in both groups showed a significant decrease in hip BMD (Table 5). The strength group showed a decrease in total BMD for CKD stages 4 and 5 and a decrease in total body T score for CKD stage 5.

**Table 5:** BMD, T score and Z score in the strength group, balance group and whole group at baseline (T0) and after 12 months of exercise training (T12) stratified for CKD stages.

	Strength group			Balance group			Whole group		
	T0	T12	*P*	T0	T12	*P*	T0	T12	*P*
Lumbar spine BMD (g/cm^2^)									
CKD 3	1.330 ± 0.379	1.345 ± 0.391	.386	1.355 ± 0.503	1.256 ± 0.368	.274	1.342 ± 0.432	1.303 ± 0.371	.145
CKD 4	1.181 ± 0.304	1.174 ± 0.308	.568	1.200 ± 0.270	1.211 ± 0.267	.471	1.191 ± 0.283	1.195 ± 0.284	.976
CKD 5	1.277 ± 0.170	1.260 ± 0.203	.460	1.069 ± 0.168	1.102 ± 0.175	.337	1.167 ± 0.195	1.172 ± 0.120	.986
Lumbar spine T score									
CKD 3	1.02 ± 3.09	1.16 ± 3.19	.324	1.26 ± 4.13	0.35 ± 2.91	.535	1.14 ± 3.54	0.78 ± 3.01	.230
CKD 4	–0.11 ± 2.48	–0.24 ± 2.46	.08	–0.06 ± 2.16	–0.07 ± 2.18	.912	–0.08 ± 2.29	–0.15 ± 2.29	.231
CKD 5	0.663 ± 1.30	0.51 ± 1.54	.433	–0.92 ± 1.39	–0.83 ± 1.45	.367	–0.22 ± 1.54	–0.23 ± 1.60	.866
Lumbar spine Z score									
CKD 3	1.33 ± 3.02	1.46 ± 3.08	.378	1.66 ± 4.06	0.76 ± 2.7	.37	1.49 ± 3.48	1.13 ± 2.89	.188
CKD 4	0.69 ± 2.06	0.63 ± 2.07	.336	0.47 ± 2.05	0.48 ± 2.09	.89	0.57 ± 2.04	0.55 ± 2.07	.690
CKD 5	1.09 ± 0.84	0.98 ± 1.14	.540	–0.43 ± 1.64	–0.24 ± 1.71	.09	0.244 ± 1.52	0.30 ± 1.57	.585
Hip BMD (g/cm^2^)									
CKD 3	1.052 ± 0.198	1.046 ± 0.193	.285	1.044 ± 0.421	1.056 ± 0.456	.542	1.048 ± 0.311	1.050 ± 0.331	.835
CKD 4	0.880 ± 0.220	0.864 ± 0.210	**.019**	0.871 ± 0.141	0.855 ± 0.139	**.007**	0.875 ± 0.178	0.859 ± 0.017	**.001**
CKD 5	0.912 ± 0 172	0.907 ± 0.156	.671	0 911 ± 0.125	0.918 ± 0.115	.548	0.912 ± 0.144	0.913 ± 0.131	.986
Hip T score									
CKD 3	–0.17 ± 1.40	–0.17 ± 1.40	1.00	–0.11 ± 2.88	–0.01 ± 3.12	.502	–0.14 ± 2.14	–0.09 ± 2.29	.530
CKD 4	–1.37 ± 1.45	–1.42 ± 1.45	.477	–1.50 ± 0.95	–1.59 ± 0.89	.461	–1.45 ± 1.19	–1.51 ± 1.17	.300
CKD 5	–1.11 ± 1.09	–1.13 ± 0.98	.888	–1.01 ± 0.90	–0.97 ± 0.87	.602	–1.06 ± 0.97	1.04 ± 0.896	.764
Hip Z score									
CKD 3	–0.37 ± 1.11	0.39 ± 1.17	.729	0.58 ± 2.86	0.68 ± 3.17	.504	0.47 ± 2.05	0.53 ± 2.25	.430
CKD 4	–0.36 ± 1.31	–0.35 ± 1.27	.744	–0.76 ± 0.90	–0.81 ± 0.83	.569	–0.59 ± 1.11	0.61 ± 1.06	.740
CKD 5	–0.38 ± 0.68	–0.35 ± 0.64	.756	–0.53 ± 0.82	–0.40 ± 0.89	.155	–0.46 ± 0.75	–0.38 ± 0.76	.173
Total body BMD (g/cm^2^)									
CKD 3	1.208 ± 0 136	1.205 ± 0 149	.683	1.194 ± 0.269	1.218 ± 0.311	.199	1.201 ± 0.206	1.212 ± 0.237	.298
CKD 4	1.131 ± 0.160	1.116 ± 0.159	**.0001**	1.161 ± 0.127	1.154 ± 0.143	.191	1.148 ± 0.143	1.137 ± 0.150	**.003**
CKD 5	1.153 ± 0.155	1.136 ± 0.156	**.026**	1.121 ± 0.094	1.111 ± 0.106	.308	1.136 ± 0.122	1.122 ± 0.127	**.035**
Total body T score									
CKD 3	0.26 ± 1.55	0.21 ± 1.68	.677	0.42 ± 2.93	0.59 ± 3.10	.243	0.40 ± 2.28	0.40 ± 2.43	.483
CKD 4	–0.62 ± 1.64	–0.74 ± 1.61	.184	–0.40 ± 1.36	–0.33 ± 1.38	.302	–0.50 ± 1.48	–0.516 ± 1.49	.787
CKD 5	–0.25 ± 1.46	–0.44 ± 1.46	**.030**	–0.47 ± 1.03	–0.21 ± 0.90	.075	–0.372 ± 1.20	–0.31 ± 1.15	.521
Total body Z score									
CKD 3	0.38 ± 1.30	0.33 ± 1.38	.699	0.66 ± 2.94	0.90 ± 3.09	.090	0.52 ± 2.21	0.62 ± 2.34	.263
CKD 4	0.01 ± 1.28	–0.09 ± 1.28	.051	0.01 ± 1.30	0.26 ± 1.22	**.028**	0.01 ± 1.28	0.11 ± 1.25	.179
CKD 5	–0.03 ± 0.94	–0.18 ± 1.05	.134	–0.24 ± 1.16	0.09 ± 1.13	.054	–0.14 ± 1.05	–0.03 ± 1.07	.288

Data are presented as mean ± SD.

A *p* value < 0.05 was accepted as the level of significance.

### Changes (Δ values) in DEXA measurements

Δ values for BMD, T and Z scores are presented in Table 6. There were significant between-group differences. Δ total body T score was negative in the strength group and unchanged in the balance group, with a statistically significant between-group difference (*P* = .005). Δ total body Z score was negative in the strength group and positive in the balance group , with a statistically significant between-group difference (*P* = .005). There were no other statistically significant between-group differences.

**Table 6:** Change (Δ score) in BMD, T score and Z score in the strength group, balance group and whole group after 12 months of exercise training.

	Strength group	Balance group	*P*	Whole group
Lumbar spine				
ΔBMD (g/cm^2^)	–0.001 ± 0.635	0.010 ± 0.051	0.344	0.005 ± 0.057
ΔT score	–0.05 ± 0.47	0.027 ± 0.40	0.357	–0.01 ± 0.44
ΔZ score	–0.01 ± 0.41	0.07 ± 0.45	0.379	0.03 ± 0.43
Hip				
ΔBMD (g/cm^2^)	0.009 ± 0.036	–0.008 ± 0.038	0.83	–0.009 ± 0.037
ΔT score	–0.02 ± 0.36	–0.01 ± 0.34	0.85	–0.02 ± 0.35
ΔZ score	0.03 ± 0.30	0.01 ± 0.47	0.73	0.02 ± 0.39
Total body				
ΔBMD (g/cm^2^)	–0.011 ± 0.023	–0.003 ± 0.038	0.18	–0.007 ± 0.032
ΔT score	–0.09 ± 0.43	0.16 ± 0.40	**0.011**	0.02 ± 0.43
ΔZ score	–0.08 ± 0.28	0.26 ± 0.58	**0.001**	0.10 ± 0.50

Data are presented as mean ± SD.

A *p* value < 0.05 was accepted as the level of significance.

### Deterioration of bone health

**Figure 4:**
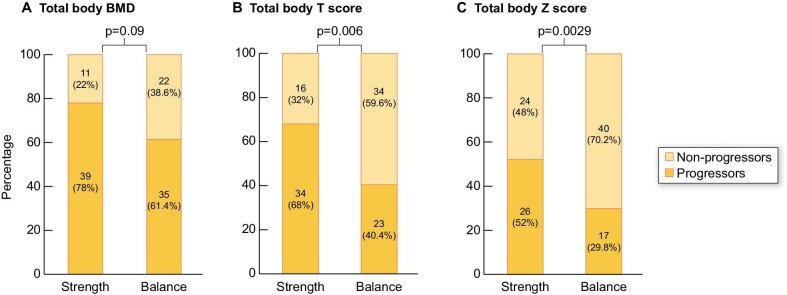
Proportion of progressors in the strength and balance groups, respectively, after 12 months of exercise training.

The proportion of progressors assessed by total body ΔT score and ΔZ score, respectively, was significantly lower in the balance group. The ΔT score showed a negative progression in 40% of the patients in the balance group compared with 68% in the strength group (*P* = .006). The ΔZ score showed a negative progression in 30% of the patients in the balance group compared with 52% of the patients in the strength group (*P* = .029) (Fig. 4).

### Univariate and multivariate logistic regression analyses

Univariate logistic regression was used to evaluate possible clinical and biochemical factors that could affect bone mass negatively. Progressors versus non-progressors were evaluated using total body ΔBMD, ΔT score and ΔZ score separately. Of the tested regressors only balance training was found to lower the risk of deterioration in total body T score (0.32, 95% CI 0.15–0.70, *P* = .005) and Z score (0.39, 95% CI 0.18–0.86, *P* = .021) but not for BMD (Tables 7 and 8). In a multivariate logistic regression analysis balance training was the only factor of statistical significance, decreasing the risk of progression of total BMD, T score and Z score.

**Table 7:** Risk of progression according to delta Δ body T score, Z score and BMD, respectively, in univariate analyses.

	T score			Z score			BMD		
	OR	CI	*P*	OR	CI	*P*	OR	CI	*P*
Balance group	**0.318**	**0.144–0.705**	**.005**	**0.392**	**0.177–0.868**	**.021**	0.449	0.191–1.056	.066
Sex	0.634	0.284–1.4	.266	0.747	0.334–1.67	.477	0.870	0.367–2.067	.753
Age	0.998	0.970–1.026	.876	0.989	0.961–1.018	.466	0.988	0.958–1.020	.466
Δ weight	1.008	0.911–1.116	.879	1.016	0.916–1.127	.761	0.946	0.844–1.060	.341
Δ BMI	0.968	0.719–1.304	.968	1.069	0.789–1.449	.665	0.813	0.580–1.14	.230
P-calcium T0	0.696	0.03–16.24	.822	3.5	0.141–88	.442	0.684	0.22–20.8	.828
P-phosphate T0	2.2	0.476–10.35	.310	1.48	0.33–6.6	.607	1.58	0.299–8.43	.587
P-PTH T0	1.004	0.982–1.026	.734	1.001	0.979–1.023	.965	1.007	0.981–1.035	.585
P-ALP T0	1.681	0.768–3.6	.194	0.95	0.608–1.488	.821	1.49	0.647–3.42	.349
P-25(OH)D T0	0.991	0.973–1.008	.293	0.988	0.970–1.006	.180	0.993	0.974–1.012	.484
P-calcium T12	0.682	0.27–16.9	.815	2.57	0.094–70	.575	1.04	0.32–34.1	.982
P-phosphate T12	0.809	0.141–4.6	.812	0.399	0.61–2.6	.339	1.18	0.175–8.019	.863
P-PTH T12	0.984	0.946–1.023	.417	0.955	0.911–1.002	.058	1.00	0.958–1.043	.993
P-ALP T12	0.944	0.801–1.112	.490	0.918	0.677–15.8	.212	0.911	0.728–1.141	.418
P-25(OH)D T12	1.002	0.987–1.017	.803	0.988	0.983–1.01	.825	1.008	0.99–1.025	.386
PPI	1.657	0.657–4.065	.270	1.010	0.415–2.45	.983	0.965	0.371–2.508	.942
Calcium-based phosphate binders	1.098	0.495–2.437	.818	0.659	0.289–1.501	.320	2.045	0.811–5.158	.129
Active vit D	0.955	0.451–2.197	.991	0.497	0.222–1.111	.088	1.838	0.787–4.292	.159
Native vit D	0.904	0.299–3.517	.886	0.725	0.171–3.07	.662	0.882	0.207–3.765	.866
Diuretics	0.686	0.302–1.558	.368	0.513	0.225–1.168	.112	0.891	0.366–2.168	.799

T0, at baseline; T12, after 12 months of training; BMI, body mass index; PTH, parathyroid hormone; ALP, alkaline phosphatase; 25(OH)D, 25 hydroxycholecalciferol; P, plasma; PPI, proton pump inhibitors; OR, odds ratio; CI, 95% confidence interval.

A *p* value < 0.05 was accepted as the level of significance.

**Table 8:** Risk of progression according to Δ total body T score, Z score and BMD, respectively, in multivariate analyses.

	T score			Z score			BMD		
	OR	CI	*P*	OR	CI	*P*	OR	CI	*P*
Balance group	**0.297**	**0.131–0.670**	**.003**	**0.367**	**0.163–0.825**	**.015**	**0.329**	**0.127–0.851**	**.022**
Sex	0.558	0.239–1.304	.178	0.698	0.302–1.611	.399	0.870	0.367–2.067	.753
Age	0995	0.966–1.025	.755	0.986	0.958–1.016	.361	0.988	0.958–1.020	.466

OR, odds ratio; CI, 95% confidence interval.

## DISCUSSION

This study's main findings were firstly positive effects of balance training on total body BMD as well as T and Z scores. Secondly, a greater proportion of participants in the strength group was classified as progressors, showing a deterioration of bone health. Thirdly, analysing the whole group, balance training emerged as a protective factor for lowering the risk of deterioration of bone health. Finally, prevalence of osteoporosis, and consequently the fracture risk, was unchanged in both groups implying positive effects on bone health.

There were no changes in BMD the spine or the hip in either group, but deterioration in the hip in the whole group and in patients with CKD stage 4 in both groups. Interpretation and clinical implications of these results are not directly apparent as lumbar spine and hip BMD are the most affirmed parameters of bone health. Moreover, previous studies in patients on dialysis have reported that decreased BMD mostly involves the hip but not the spine [21, 22]. As this study did not comprise a usual care group and there are no observational studies in patients with CKD stages 3 to 5, it is impossible to assess whether exercise training attenuated progression. In an observational study in patients on haemodialysis, not participating in any exercise training, Malluche *et al*. reported a 1.2% and 3.1% decline in BMD in the hip after 1 and 2 years, respectively [22]. In our interventional study we found a decrease of 1% and 0.6% in the hip and total body BMD, respectively, after 12 months of exercise training, which might indicate a positive effect of exercise training in slowing a deterioration of bone health.

Different types of physical exercise have been reported to potentially stimulate osteocytes and influence bone formation and resorption [23, 24]. There is substantial evidence supporting the positive effects of exercise interventions on bone health in different age groups in the general population as well as in patients with primary osteoporosis [25–27]. Although exercise training as a non-pharmacological strategy for the maintenance of bone health is widely recognized, the impact of exercise training on bone parameters in patients with CKD is less well established. The first systematic review on the topic, analysing observational and experimental studies on bone outcomes after exercise training in patients with CKD, was published in 2020 by Cardoso *et al*. [25]. Thirteen studies (six observational and seven interventional) were found and included in the analysis. Apart from one exception, all studies in the review analysed various forms of exercise training on a single effect of bone health parameters in patients on KRT. Only one report studied the effects of aerobic exercise on markers of bone metabolism in obese patients with CKD not on KRT [28]. These studies show conflicting results. Three of six observational studies included in the systematic review suggest a positive association between exercise training and BMD in the femoral neck and lumbar spine in patients on haemodialysis and on total body BMD both in patients on dialysis and with a kidney transplant. The interventional studies [five randomized controlled trials (RCT) and two non-RCT] had relatively small samples sizes (13–52 subjects) and most participants were on haemodialysis. The most common type of exercise training was resistance training usually during the dialysis session [25]. Differences in type of KRT as well as different exercise methods and durations (2–24 weeks) do not allow a direct comparison with the present study comprising patients with CKD stages 3–5 not on KRT with 12 months of exercise training.

In the primary analysis of the RENEXC trial 12 months of either strength or balance training, both combined with endurance training, improved or maintained overall endurance, muscular endurance and strength and balance [12]. In this prespecified sub-study, we found that only the balance group showed significant positive results regarding bone health parameters. Possible explanations could be that the balance exercises prescribed were performed in a standing position which increases skeletal load and focused on engaging muscles in the legs and trunk like standing on one leg, tandem stance and single-leg deadlift. The strength exercises on the other hand were mainly performed in a sitting or lying position, predominately involving the arms and legs, like quadriceps extension, push-ups and biceps curls. Both groups were prescribed endurance training, like walking, jogging and cycling, which also focused on leg muscles. As has been reported previously, patients in the balance group reported more minutes of endurance training per week than patients in the strength group, thus further increasing the impact on leg and trunk muscles [12, 29].

While current clinical practice guidelines for the prevention and management of osteoporosis recommend exercise training as an effective approach to maintain bone mass or slow bone loss throughout the postmenopausal years and into old age, the optimal exercise training program to prevent osteoporosis and osteoporosis related fractures has yet to be determined [25, 30].

Progressive resistance training is recommended to increase or maintain BMD in postmenopausal women. There is a growing body of evidence supporting the role of multimodal programs that incorporate weight-bearing impact-loading activities, progressive resistance exercises targeting muscles attached to or crossing the hip and spine and functionally challenging balance and mobility activities [29]. Results from our study are congruent with the findings that exercises involving the legs and trunk, targeting the above-mentioned muscles and challenging balance and mobility may have beneficial effects in preserving BMD. It seems reasonable to assume that skeletal load or strain seems to be a pivotal factor for enhancing bone health, thus, suggesting that balance training together with endurance training might be the preferred training modality for preventing bone loss in combination with improving balance in patients with CKD. Improved balance is of special interest considering the high risk of falls in the elderly and frail CKD population [12].

There are several strengths in this study. Firstly, the participants represent a typical cohort of patients with CKD stages 3–5 not on KRT, as the majority are elderly and have several comorbidities. Secondly, the duration of the interventional period was 12 months, which was positive with respect to time needed to influence bone and detect changes with DEXA. Finally, to our knowledge, this is the first study to compare effects of strength versus balance training on bone health parameters in patients with CKD not on KRT. This study also has limitations, especially the absence of a sedentary control group, but the reasons for that have been discussed and explained previously [12]. A longer observation period might have revealed more skeletal effects of the two exercise regimes and could have had specific effects on the hip and lumbar spine. Moreover, trabecular bone score was not assessed.

In conclusion, the prevalence of osteoporosis and osteopenia was unchanged after 12 months of exercise training with no between-group differences. Exercises performed in the standing position engaging the whole body and core muscles, as performed by the balance group, together with endurance training, seemed to be superior in maintaining and improving whole body BMD compared with strength training, performed in a sitting or lying position, together with endurance training. Thus, balance training emerges as an important component of exercise training in patients with CKD stages 3–5 not on KRT.

## Data Availability

Data available on request.
